# Multi-Center Evaluation of Post-Operative Morbidity and Mortality after Optimal Cytoreductive Surgery for Advanced Ovarian Cancer

**DOI:** 10.1371/journal.pone.0039415

**Published:** 2012-07-23

**Authors:** Arash Rafii, Eberhard Stoeckle, Mehdi Jean-Laurent, Gwenael Ferron, Philippe Morice, Gilles Houvenaeghel, Fabrice Lecuru, Eric Leblanc, Denis Querleu

**Affiliations:** 1 Institut Claudius Regaud, Toulouse, France; 2 Institut Bergonié, Bordeaux, France; 3 Institut Gustave Roussy, Paris, France; 4 Institut Paoli Calmettes, Marseille, France; 5 Hôpital Européen Georges Pompidou, Paris, France; 6 Centre Oscar Lambret, Lille, France; University Clinic of Navarra, Spain

## Abstract

**Purpose:**

While optimal cytoreduction is the standard of care for advanced ovarian cancer, the related post-operative morbidity has not been clearly documented outside pioneering centers. Indeed most of the studies are monocentric with inclusions over several years inducing heterogeneity in techniques and goals of surgery. We assessed the morbidity of optimal cytoreduction surgery for advanced ovarian cancer within a short inclusion period in 6 referral centers dedicated to achieve complete cytoreduction.

**Patients and Methods:**

The 30 last optimal debulking surgeries of 6 cancer centers were included. Inclusion criteria included: stage IIIc- IV ovarian cancer and optimal surgery performed at the site of inclusion. All post-operative complications within 30 days of surgery were recorded and graded using the Memorial secondary events grading system. Student-t, Chi2 and non-parametric statistical tests were performed.

**Results:**

180 patients were included. There was no demographic differences between the centers. 63 patients underwent surgery including intestinal resections (58 recto-sigmoid resection), 24 diaphragmatic resections, 17 splenectomies. 61 patients presented complications; One patient died post-operatively. Major (grade 3–5) complications requiring subsequent surgeries occurred in 21 patients (11.5%). 76% of patients with a major complication had undergone an ultraradical surgery (P = 0.004).

**Conclusion:**

While ultraradical surgery may result in complete resection of peritoneal disease in advanced ovarian cancer, the associated complication rate is not negligible. Patients should be carefully evaluated and the timing of their surgery optimized in order to avoid major complications.

## Introduction

Residual disease after surgery is the main prognostic factor in advanced ovarian cancer [1]. As a consequence, cytoreductive surgery completed by platinum-based chemotherapy is the mainstay of treatment in this setting, even though it remains unclear whether the correlation between cytoreduction and outcome is related to treatment, to tumor biology, or to both. What is ascertained is the lack of relevance of suboptimal debulking which may even be harmful [2]. The definition of optimal debulking has evolved over time. Gynecology Oncology Group (GOG) criteria are residual nodules <1 cm [3], but current opinion tends to consider only complete macroscopic resection as optimal [4]–[7]. For surgeons convinced of the therapeutic impact of cytoreduction, it may be tempting to increase the surgical effort to achieve higher rates of optimal resections. Thus single series studies have reported high survival rates with radical surgeries [8]. Optimal surgery can also be achieved by performing the cytoreductive surgery after neo-adjuvant chemotherapy enhancing the likelihood to obtain complete cytoreduction during interval debulking surgery [9]. In the EORTC/Gynecologic Cancer Intergroup randomized trial comparing neo-adjuvant to standard treatment with primary surgery, survival was found to be similar in both groups, but morbidity was lower for patients who underwent interval debulking surgery [9]. Debate is ongoing whether to prefer standard or neo-adjuvant treatment [10]. Morbidity may be an important factor for decision taking. Chi et al. have demonstrated that the use of extensive upper abdominal surgical procedures significantly increased the rate of optimal primary cytoreduction without significant increase in post-operative complications [8]. More recently they reported major complication rate of 22% (grade 3–5) in patients with extensive upper abdominal surgical procedures [11].

Extensive debulking surgery may increase morbidity and delay initiation of chemotherapy [11]. Collecting data of post-operative complications by an external observer in several centers may give better insight in such issue than monocentric studies. In this study we collected and analyzed morbidity data in patients who underwent optimal debulking surgeries throughout six institutions recognized as referral centers in the treatment of ovarian cancer in France.

## Methods

The following specialized and high-volume care centers were included in this longitudinal retrospective study.

Institut Claudius Regaud, Toulouse; Institut Gustave Roussy, Villejuif; Institut Paoli Calmettes, Marseille; Hôpital Européen Georges Pompidou, Paris; Centre Oscar Lambret, Lille; Institut Bergonié, Bordeaux.

In order to avoid any bias due to case selection we included the last 30 patients who underwent complete cytoreductive surgery for an advanced ovarian cancer in each center.

All surgeries were performed by/or under the supervision of a senior surgeon (more than 5 years of experience in the management of advanced ovarian cancers).

Inclusion criteria: we considered patients with primary epithelial ovarian carcinoma stages IIIC and IV operated in the participating centers. Stage IIIC disease included patients with bulky peritoneal disease, but not those with lymph node involvement only. All surgical procedures had to be performed in the inclusion center, achieving complete (residual tumor less than 2 mm) cytoreductive surgery. In case of neo-adjuvant chemotherapy, diagnosis and tumor extent was assessed by an initial laparoscopy.

Primary debulking surgery (PDS) was defined as patients undergoing a debulking surgery before any chemotherapeutic treatment. Interval debulking surgeries (IDS) was defined as patient undergoing a debulking surgery after cycles of neo-adjuvant treatment. The numbers of cycles were at the discretion of the treating physician.

### Ethics Statement

All data were recorded without identifiers therefore our study did not require informed consent from patients. The need for written informed consent from the participants was waived as this was an audit with no identifiers accessible to the external observers.

### Patient Chart Collection and Definitions

All patients’ charts were collected and analyzed by external observers (AR, JLM) according to a predefined checklist. Demographic, per and postoperative data were recorded. Sugarbaker scoring system [12] was used to describe the extent of disease at the beginning of the cytoreductive surgery. The surgeries were classified as “standard” or “radical”. Standard surgery included: total abdominal hysterectomy, bilateral salpingo-oophorectomy, omentectomy, appendectomy, peritonectomies involving the pelvis, and pelvic and para-aortic lymphadenectomies. Radical surgery included standard surgery with any of the following procedures: bowel resection, splenectomy, caudal pancreatectomy, large stripping of the peritoneum removing more than 5 cm^2^, liver resection.

The Memorial Sloan-Kettering Cancer Center surgical secondary events grading system [13] was used to assess complication rate. Grade 1–2 complications were considered as minor and grade 3–5 complications as major. For patients with more than one complication, only the highest-grade complication was considered for the analysis.

We considered all events occurring within 30 days after debulking surgery.

All patients were staged according to the International Federation of Gynecology and Obstetrics (FIGO) system [14].

### Statistical Analysis

Categorical variables were compared using Fisher exact test and continuous variables were compared using the Student t-test. All statistical tests were two-sided and differences were considered significant at a level of p<0.05.

## Results

180 patients operated between 2005 and 2008 were included. Demographic characteristics of the population are presented in Table 1, most of the patients had an American Society of Anesthesiologists (ASA) score of 2 to 3 (81,7%). 109 patients presented with ascites at the time of diagnosis with a mean volume of 930,1 ml. 72 patients (40%) presented with anemia and 22 patients (12.2%) had hypo-albuminemia at the time of diagnosis (albumin was considered low below 30 g/l). Most patients had serous adenocarcinoma and grade 3 disease; 25 patients (13,8%) had stage IV disease (Table 2).

**Table 1:** Demographic characteristics of the patients.

***Mean age (+/− SD)***	61,5+−11.5 years (20–83)
***BMI***	24,2+/−5.1 (15,5–55,5)
***ASA***	
1	32 (18,8%)
2	114 (64,7%)
3	30 (17%)
***Past Medical History***	
Diabetes	8 (4,4%)
Hypertension	48 (26,6%)
Tabacco	12 (6,6%)
Vascular disease	15 (8.3%)
Others	63 (35%)
***Past Medical surgeries***	
Mid-line Laparotomies	23 (12.7%)
Other Lapartomies	67 (37.2%)
***Ascitis***	109 patients (60.5%)
	mean volume 930.11 mL (0–600)
***Biological Parameters***	
CA 125 mU/L	1347 UI/L (10–21000)
Pre-operative Hb g/100 ml	11,7+/−1.3 (8–15.3)
Pre-operative Platelets/mm3	322 201 (80000–1077000)
Pre-operative Albumin g/l	36,5 = /−8 (13–47)

**Table 2:** Surgical and tumor characteristics.

***FIGO stage***	
IIIc	155 (86,1%)
IV	25 (13,8%)
***Sugarbaker score***	14.8 (10–33)
***Histologic subtype***	
Serous	147 (82%)
Mucinous	5 (2,9%)
Endometrïode	7 (3,7%)
Mixte	2 (1.4%)
Clear cell Carcinoma	7 (3.7%)
Non- differentiated	11 (6.3%)
Tubuleux	1 (0.7%)
***Grade***	
1	11 (6.2%)
2	47 (26.3%)
3	122 (68%)

**Figure 1:**
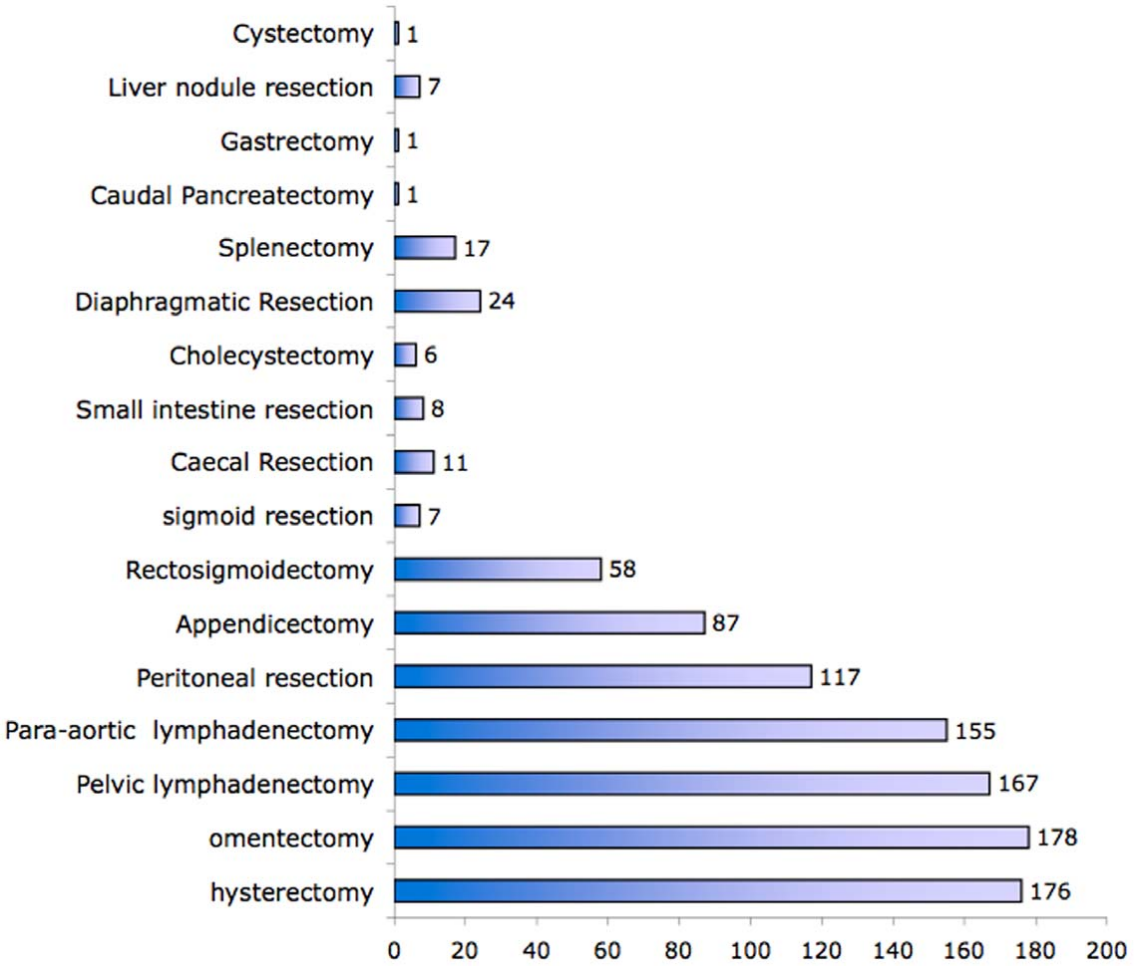
Surgical procedures performed.

The mean Sugarbaker score was 14.8, ranging from 10 to 33. Sixty (33.3%) patients had an extensive disease with Sugarbaker score above 10. 128 (71%) patients underwent a laparoscopy before the cytoreductive surgery. Seventy one patients (40%) underwent PDS followed by adjuvant chemotherapy. One hundred and nine patients (60%) received neo-adjuvant chemotherapy and underwent IDS after a mean of 3.2 cycles of neoadjuvant chemotherapy. There were no demographic differences between these two groups. The procedures performed are depicted in Figure 1. Ninety five patients (52.7%) underwent standard surgery and 85 patients (48%) radical surgery. Mean hospital stay was 13,7+/−9.7 days; mean surgical intensive care unit (SICU) duration was 3.4+/−5.1 days. Most patients resumed bowel function within 3.2 days (Table 3).

**Table 3:** Post-operative parameters.

**Hospital Stay (Days)**	13.9+/−9.7 (5–100)
**SICU Stay (Days)**	3.4+/−5.1 (0–37)
**Bowel function**	3.2 jours +/−4 (0–30)
**Complications**	61 patients (33%)
Minor (grade 1–2)	40 (22%)
Major (grade 3–5)	21 (11%)
**Morbidity Grade**	
0	119 (66.1%)
1	26 (14,4%)
2	14 (7.7%)
3	15 (8.3%)
4	5 (2,7%)
5	1 (0.5%)

There were 61 (33%) complications comprising 40 (22%) minor and 21 (11%) major complications (Table 3). One hundred twenty two patients had complete surgeries and 58 had optimal surgeries. All the patients with optimal surgeries had millimetric residual disease (bowel or mesenteric nodules that were coagulated). Only one patient included in the study died within 30 days after surgery. She presented necrotizing pancreatitis at post-operative day 2 and died of multi-organ failure. Details of complications and their treatments are presented in Table 4. 30 patients had infectious complications (local or general), 5 patients had hemorrhagic complications. Among the 21 major complications 19 (90%) required at least another surgical procedure. Overall 6 patients where managed by interventional radiology for pelvic abscesses or lymphoceles.

**Table 4:** Complications and their management.

	Total	Treatment		
		Medical	Radiologic	Surgical
Peritonitis	5	0	0	5
Pelvic Abcess	7	2	2	3
Sus-Mesocolic Abcess	6	4	0	2
Parietal Abcess	5	5	0	0
Sepsis	5	5	0	0
UTI	2	2	0	0
Fistula	4	0	0	4
Hemoperitoineum	3	0	0	3
Intra-abdominal Hematoma	2	0	0	2
Seroma formation	14	9	4	1
Bowel Obstruction	2	0	0	2
Functional ileus	5	5	0	0
Pleuresia	6	6	0	0
Pancreatitis	1	0	0	1

We analyzed the different factors involved in the occurrence of complications. ASA score, presence of ascitis and previous surgeries were not associated to the occurrence of complications (overall or major only) in univariate analysis.

**Figure 2:**
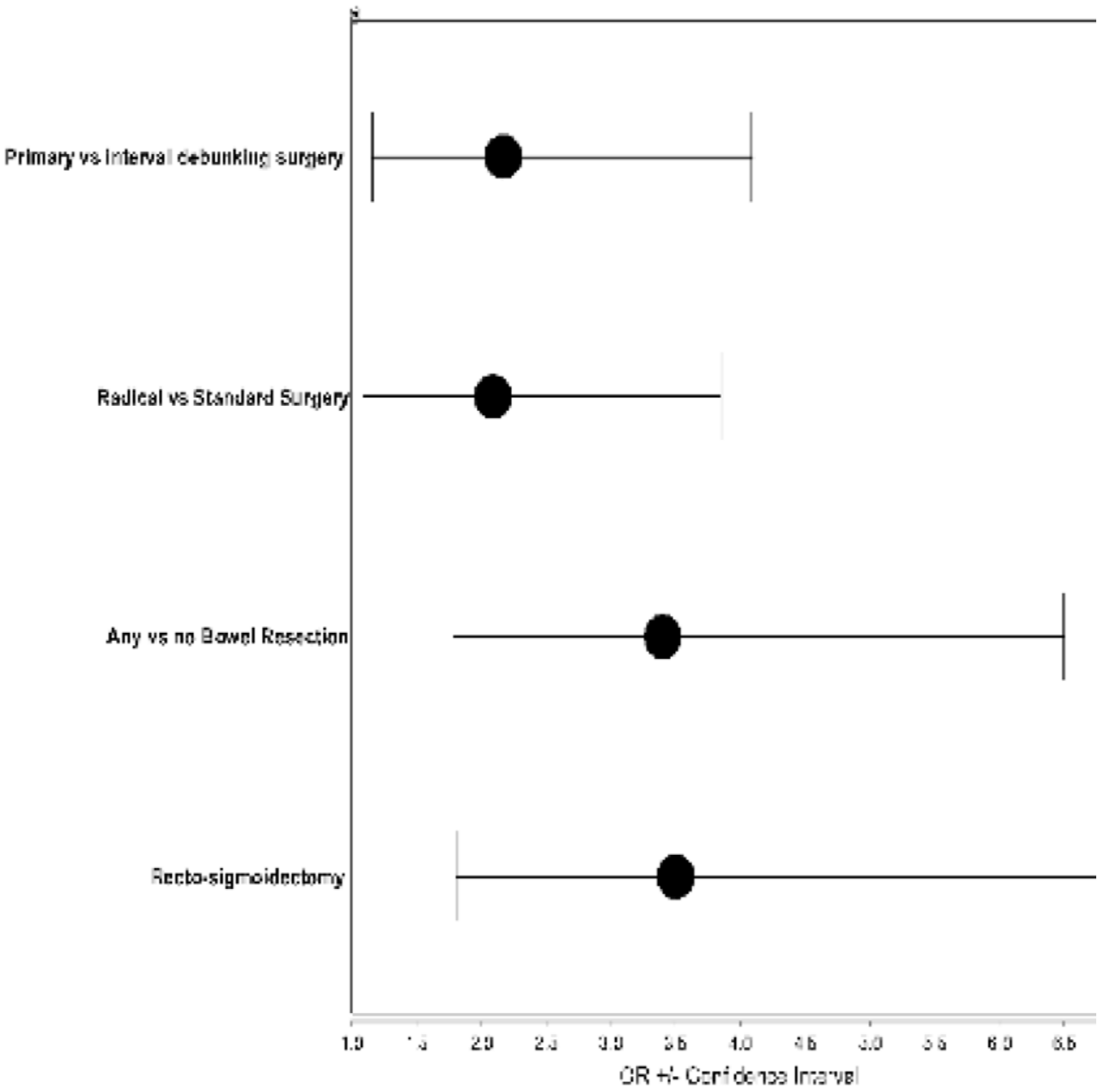
Odd ratios for different parameters associated to occurrence of complications.

As demonstrated in Table 5 there were no significant differences in preoperative initial Sugarbaker score between the complicated and uncomplicated groups of patients. The rate of complications was higher in patients with PDS compared to those having IDS (OR 2.17 (1.16–4.09)). Patients who underwent radical surgery had an increased risk of complication compared to patients who underwent standard surgery (OR 2.05 (1.09–3.85)). Among the procedures, performing any bowel resection was associated with increased complications (OR 3.4 (1.78–6.5)). Recto-sigmoidectomy was in particular associated with an increased risk of complications (OR 3.5, (1.81–6.81)) (Table 6, Figure 2). Sugarbaker score of the patients with bowel resections who presented major complications was significantly higher than peritoneal carcinosis index of patients with bowel resection without major complications (19+/−5.03 versus 10+/−6.32).

**Table 5:** Univariate analysis of factors associated with complications.

Parameters	No complications (N = 119)	Overall Complications (N = 61)	p	Major Complications (N = 21)	p
**Age**	61.2 (+/−11.2)	61.6 (+/−12.4)	0.71	62.5 (+/−11.7)	0.7
**BMI**	23.3 (+/−4.9)	24.8 (+/−5.4)	0.32	27 (+/−4)	0.32
**Sugarbaker Score**	7.36 (+/−5.9)	9.01 (+/−6.5)	0.089	14 (+/−4)	0.02
**ASA Score**					
**1**	15 (12,5%)	18 (30%)		7 (33%)	
**2**	82 (68,3%)	32 (53,3%)	0.017	6 (28%)	0.017
**3**	19 (15,8%)	12 (20%)		8 (38%)	
**Up-Front Surgery** **(71 patients)**	40 (33%)	31 (51%)	0.01	11 (52%)	0.01
**Hb**	11.41 (n = 99)	11.54 (n = 54)	0.7388	11 (n = 21)	0.7388

**Table 6:** Association between procedures and occurrence of a complication.

Surgical Parameters	No complications	Overall	*P*	Major	*p*
	(N = 119)	Complications (N = 61)		Complications (n = 21)	
**Surgery Type**					
Standard	70 ( 59%)	25 (40.9%)		5 (23,8%)	
Radical	49 (41%)	36 (59%)	*0.02*	16 (76,2%)	*0.013*
**Procedures**					
Any Bowel Resection	29 (24%)	32 (52.4%)	*0.0005*	14 (66.66%)	*0.0005*
Recto-sigmoïdectomy	27 (22.5%)	31 (50%)	*0.0002*	14 (66.66%)	*0.0002*
Left Hemi-colectomy	1 (0.84%)	6 (9.8%)	*0.003*	0	*0.0033*
Right Hemi-colectomy	4 (3.3%)	7 (11.4%)	*0.03*	3 (14.3%)	*0.0328*
Small Bowel resection	1 (0.84%)	6 (9.8%)	*0.578*	18 (85.7%)	*0.578*
Diaphargmatic resection	16 (13.4%)	8 (13.1%)	*0.9*	4 (19%)	*0.007*
Surgery Duration (mn)	316.7 (79.6)	325.6 (88.3)	*0.52*	329	*0.52*
Drain (numbers)	1.4 (+/- 0.9)	1.2 (+/- 0.9)	*0.28*	1.4 (+/_0.8)	*0.29*
**Blood Loss (ml)**	1342.07 (n = 65)	1900.4 (n = 45)	*0.06*	2263 (+/-1379)	*0.0099*

Diaphragmatic and small bowel resections were not associated with increased complications. Operative parameters such as duration of surgery, results of the surgery (optimal versus complete) and use of drains were not associated with post-operative complications on univariate analysis.

## Discussion

In this multicentric analysis of surgical morbidity during cytoreductive surgery for advanced ovarian cancer we identified the following variables being predictive for complications: timing of surgery (PDS vs. IDS), the extent of surgery (radical vs. standard) and realization of a colic or rectal resections. This is concordant with a recent study published by Aletti et al. [15]. By studying the outcome for 576 patients with stage IIIC or IV they identified a subgroup of patients characterized by high initial tumor dissemination, poor performance or nutritional status and age ≥75. In this group, high surgical effort to achieve low residual disease was associated with morbidity of 63.6% and limited survival benefit.

We used the MSKCC scoring system to classify complications occurring in our patients. Our overall rate of complications was 33% (61 patients/180 patients), with 11% (22 patients/180 patients) having severe complications. The complication rate in a meta-analysis using population-based reports varied between 2.5% to 4.8% [16]. Single center studies displayed even lower complication rate around 2.5% which might not reflect the overall complication rate in a multicenter setting [16]. There are several bias to such reports including retrospective bias as well as reporting bias. Retrospective studies for example induce heterogeneity both in the population and treatment modalities.

We chose a different approach and established an independent audit. We selected 6 different reference centers for the treatment of advanced ovarian carcinomas. In order to avoid selection bias we performed a longitudinal study including the 30 last patients who underwent optimal debulking surgery for ovarian cancer. Two external reviewers independently reviewed the records. The patients were homogenous with no significant demographic differences across the six centers. We included only the last 30 patients per center to reduce as much as possible the bias induced by heterogeneity of care induced by long inclusion periods. Therefore we have limited most of the bias induced by retrospective studies. Several studies have documented that gynecologist-oncologist usually gives optimal level of care in advanced ovarian cancers [17]. As we selected 6 reference centers, we ensured that all patients included in this study were managed by a gynecologist-oncologist.

The inclusion criteria of this study was complete surgery achieved we did not record the number of patients in which complete surgery was attempted. However in multicentric studies performed in France including many of the centers which participated in this study, the rate of complete surgery was around 70%–85% [18].

As demonstrated by the univariate and multivariate analysis, most of the major complications were related to bowel resections (14/22).

Other radical procedures such as extensive peritoneal resections or diaphragmatic resections were not associated to significant increase of major complications. This is in accordance with Chereau et al., who found an acceptable complication rate of diaphragmatic surgery for stage III/IV ovarian cancer surgeries [19]. In another study Dowdy et al. found an increase rate of pleural effusion requiring up to 12.5% of thoracocentesis however no other major complications were associated to diaphragmatic resection [20].

All patients in our study had immediate re-anastomosis. 13 patients (7.2%) had diverting protective stomas, two were performed for the management of a complication during reoperation.(one presented a pelvic abscess and another one a recto-vaginal fistula). Most of the anastomosis were performed mechanically (49/61).

Mourton et al. published their experience of 70 patients with recto-sigmoid resection and primary anastomosis [21]. The rate of protective ileostomy was higher in their study 17% with only one patient requiring reoperation for colostomy. None of the patient with a protective stoma had a complication related to anastomosis leakage. Richardson et al studied 177 patients without diverting stomas and found an anastomotic leak rate of 6.8%. The only risk factor identified in their study was low serum albumin. This low anastomotic leak rate was also reported by others ranging from 0 to 4% depending the studies [22].

Our overall leakage related complications were slightly above the range of the literature with 14/180 patients (7.7%) with a lower rate of protective stomas. The rate of complications due to anastomotic leak was 22% (14/61) if we only consider patients with bowel resection. Several factors can explain higher complication rate related to bowel resection in our study: (i) most of the patients had advanced disease with important tumor burden (ii) we only included patients with optimal surgeries and maximal surgical efforts. (iii) there is no reporting bias as external auditors longitudinally included all cases. (iv) finally the low rate of protective stoma might explain why a significant number of the anastomotic leaks directly lead to complications requiring radiological or surgical intervention. Whether we should perform diverting protective stoma in case of bowel (recto-sigmoid) resection in advanced ovarian cancer surgery cannot be answered by our study.

We were able to identify some risk factors of anastomotic leak. First Sugarbaker scores of patients with bowel resections who presented major complications were significantly higher than peritoneal carcinosis index of patients with bowel resection without major complications (19+/−5.03 versus 10+/−6.32). It seems that increased morbidity is not due to a unique surgical procedure but to accumulation of multiple bowel resections associated to extensive peritoneal resections that might act as a protective barrier. We also observed a trend for patients who had neo-adjuvant chemotherapy to develop less major complications following bowel resections (13% for patient with neo-adjuvant chemotherapy versus 32% for patients without neo-adjuvant chemotherapy). We had recorded characteristics for bowel anastomosis. While most of them were made automatically with few protective stomas the numbers of complications did not allow us to carry an insightful statistical analysis. In the participating centers the bowel resection and anastomosis was directly made by the gynecologist-oncologists (while most of the Gynecologist-oncologists had a digestive surgery training, a digestive surgeons was not systematically involved to carry on procedures related to bowel resections).

A meta-analysis by Bristow et al. demonstrated that each cycle of neo-adjuvant chemotherapy will have a negative incidence on the overall survival. However a recent meta-analysis demonstrated than neoadjuvant chemotherapy helped the gynecologic oncologist achieve an increased rate of optimal cytoreduction [23], [24].

Our study demonstrates a lower number of complications in patients with neoadjuvant chemotherapy, in relation to the reduction of the need for radical surgery. The recently EORTC randomized trial has demonstrated that neo-adjuvant chemotherapy might be as good as upfront surgery in advanced ovarian cancer [9]. However this trial must be interpreted with caution as only 20.4% of the patients had a complete debulking surgery in the primary debulking surgery group. Therefore data still supports upfront surgery when optimal cytoreduction can be achieved with acceptable complication rate; neo-adjuvant chemotherapy might benefit patients presenting with extensive disease requiring radical procedures. Careful systematic laparoscopic evaluation of patients with advanced ovarian carcinoma might be a solution to determine the best management protocol for each patient. Chereau et al. demonstrated a strong association between the occurrence of postoperative complications and Aletti peritoneal cancer index, or Eisenkop scores [25].

This is a multicentric study of post-operative complications in optimal surgery of advanced ovarian carcinomas performed as an independent audit. While the overall complication rate is acceptable and justifies active surgical approach we have been able to point out bowel resection as the main cause of major complications and therefore suggest that patients requiring such procedure to be clearly identified and optimal preventive procedures applied to prevent occurrence of major complications.

## References

[1] Schwartz PE (2008). Cytoreductive surgery in the management of ovarian cancer.. Oncology.

[2] Cannistra SA, Bast RC, Berek JS, Bookman MA, Crum CP (2003). Progress in the management of gynecologic cancer: consensus summary statement. J Clin Oncol.. 15;21.

[3] Omura GA, Brady MF, Homesley HD, Yordan E, Major FJ (1991). Long-term follow-up and prognostic factor analysis in advanced ovarian carcinoma: the Gynecologic Oncology Group experience.. J Clin Oncol.

[4] du Bois A, Reuss A, Pujade-Lauraine E, Harter P, Ray-Coquard I (2009). Role of surgical outcome as prognostic factor in advanced epithelial ovarian cancer: a combined exploratory analysis of 3 prospectively randomized phase 3 multicenter trials: by the Arbeitsgemeinschaft Gynaekologische Onkologie Studiengruppe Ovarialkarzinom (AGO-OVAR) and the Groupe d’Investigateurs Nationaux Pour les Etudes des Cancers de l’Ovaire (GINECO).. Cancer 15.

[5] Peiretti M, Zanagnolo V, Aletti GD, Bocciolone L, Colombo N (2010). Role of maximal primary cytoreductive surgery in patients with advanced epithelial ovarian and tubal cancer: Surgical and oncological outcomes. Single institution experience.. Gynecol Oncol.

[6] Chi DS, Eisenhauer EL, Lang J, Huh J, Haddad L (2006). What is the optimal goal of primary cytoreductive surgery for bulky stage IIIC epithelial ovarian carcinoma (EOC)?. Gynecol Oncol.

[7] Stuart GC, Kitchener H, Bacon M, duBois A, Friedlander M (2011). 2010 Gynecologic Cancer InterGroup (GCIG) consensus statement on clinical trials in ovarian cancer: report from the Fourth Ovarian Cancer Consensus Conference. Int J Gynecol Cancer..

[8] Chi DS, Eisenhauer EL, Zivanovic O, Sonoda Y, Abu-Rustum NR (2009). Improved progression-free and overall survival in advanced ovarian cancer as a result of a change in surgical paradigm.. Gynecol Oncol.

[9] Vergote I, Tropé CG, Amant F, Kristensen GB, Ehlen T (2010). Neoadjuvant chemotherapy or primary surgery in stage IIIC or IV ovarian cancer.. N Engl J Med.

[10] Chi DS, Schwartz PE (2008). Cytoreduction vs. neoadjuvant chemotherapy for ovarian cancer.. Gynecol Oncol.

[11] Chi DS, Zivanovic O, Levinson KL, Kolev V, Huh J (2010). The incidence of major complications after the performance of extensive upper abdominal surgical procedures during primary cytoreduction of advanced ovarian, tubal, and peritoneal carcinomas.. Gynecol Oncol.

[12] Look M, Chang D, Sugarbaker PH (2003). Long-term results of cytoreductive surgery for advanced and recurrent epithelial ovarian cancers and papillary serous carcinoma of the peritoneum.. Int J Gynecol Cancer.

[13] Chi DS, Abu-Rustum NR, Sonoda Y, Awtrey C, Hummer A (2004). Ten-year experience with laparoscopy on a gynecologic oncology service: analysis of risk factors for complications and conversion to laparotomy.. Am J Obstet Gynecol.

[14] Benedet JL, Bender H, Jones H 3rd, Ngan HY, Pecorelli S (2000). FIGO staging classifications and clinical practice guidelines in the management of gynecologic cancers. FIGO Committee on Gynecologic Oncology.. Int J Gynaecol Obstet.

[15] Aletti GD, Eisenhauer EL, Santillan A, Axtell A, Aletti G (2010). Identification of patient groups at highest risk from traditional approach to ovarian cancer treatment.. Gynecol Oncol.

[16] Gerestein CG, Damhuis RA, Burger CW, Kooi GS (2009). Postoperative mortality after primary cytoreductive surgery for advanced stage epithelial ovarian cancer: a systematic review.. Gynecol Oncol.

[17] Mercado C, Zingmond D, Karlan BY, Sekaris E, Gross J (2010). Quality of care in advanced ovarian cancer: the importance of provider specialty.Gynecol Oncol..

[18] Chéreau E, Rouzier R, Gouy S, Ferron G, Narducci F (2011). 19. Morbidity of diaphragmatic surgery for advanced ovarian cancer: retrospective study of 148 cases. Eur J Surg Oncol..

[19] Chereau E, Ballester M, Selle F, Cortez A, Pomel C (2009). Pulmonary morbidity of diaphragmatic surgery for stage III/IV ovarian cancer.. BJOG.

[20] Dowdy SC, Loewen RT, Aletti G, Feitoza SS, Cliby W (2008). Assessment of outcomes and morbidity following diaphragmatic peritonectomy for women with ovarian carcinoma. Gynecol Oncol..

[21] Mourton SM, Temple LK, Abu-Rustum NR, Gemignani ML, Sonoda Y (2005). Morbidity of rectosigmoid resection and primary anastomosis in patients undergoing primary cytoreductive surgery for advanced epithelial ovarian cancer.. Gynecol Oncol.

[22] Richardson DL, Mariani A, Cliby WA (2006). Risk factors for anastomotic leak after recto-sigmoid resection for ovarian cancer.. Gynecol Oncol.

[23] Bristow RE, Chi DS (2006). Platinum-based neoadjuvant chemotherapy and interval surgical cytoreduction for advanced ovarian cancer: a meta-analysis.. Gynecol Oncol.

[24] Kang S, Nam BH (2009). Does neoadjuvant chemotherapy increase optimal cytoreduction rate in advanced ovarian cancer? Meta-analysis of 21 studies.. Ann Surg Oncol.

[25] Chereau E, Ballester M, Selle F, Cortez A, Darai E, Rouzier R (2010). Comparison of peritoneal carcinomatosis scoring methods in predicting resectability and prognosis in advanced ovarian cancer.. Am J Obstet Gynecol 202: 178 e1–178 e10.

